# High Prevalence of Malaria Parasitemia and Anemia among Hospitalized Children in Rakai, Uganda

**DOI:** 10.1371/journal.pone.0082455

**Published:** 2013-12-17

**Authors:** Valerian L. Kiggundu, Wendy P. O'Meara, Richard Musoke, Fred K. Nalugoda, Godfrey Kigozi, Enos Baghendaghe, Tom Lutalo, Marion K. Achienge, Steven J. Reynolds, Fred Makumbi, David Serwadda, Ronald H. Gray, Kara K. Wools-Kaloustian

**Affiliations:** 1 Rakai Health Sciences Program, Kalisizo-Station, Kalisizo, Rakai, Uganda; 2 Department of Medicine, Duke University School of Medicine and Duke Global Health Institute, Durham North Carolina, United States of America; 3 East African IeDEA (International Epidemiological Data Bases to Evaluate AIDS) and Infectious Diseases Institute Makerere University College of Health Sciences, Mulago Hospital Complex, Kampala, Uganda; 4 Division of Intramural Research, National Institute of Allergy and Infectious Diseases, National Institutes of Health, Bethesda, Maryland, United States of America; 5 Makerere University School of Public Health, Kampala, Uganda; 6 Department of Epidemiology, Johns Hopkins Bloomberg School of Public Health, Baltimore Maryland, United States of America; 7 Department of Medicine, Division of Infectious Diseases, Indiana University School of Medicine, Indianapolis, Indiana, United States of America; Université Pierre et Marie Curie, France

## Abstract

**Background:**

There is a paucity of data on malaria among hospitalized children in malaria endemic areas. We determined the prevalence, presentation and treatment outcomes of malaria and anemia among children in two hospitals in Rakai, Uganda.

**Methods:**

Children under five years hospitalized in Kalisizo hospital or Bikira health center in Rakai district, Uganda between May 2011 and May 2012 were enrolled and followed-up until discharge, death or referral. Data were collected on social-demographic characteristics, current and past illnesses and clinical signs and symptoms. Blood smears, hemoglobin (Hgb) levels and HIV testing were performed from finger/heel prick blood. The associations between malaria infection and other factors were estimated using log-binomial regression to estimate adjusted prevalence risk ratios (aPRR) and 95% confidence intervals (CIs), controlling for clustering at health facilities.

**Results:**

2471 children were enrolled. The most common medical presentations were fever (96.2%), cough (61.7%), vomiting (44.2%), diarrhea (20.8%), and seizures (16.0%). The prevalence of malaria parasitemia was 54.6%. Children with malaria were more likely to present with a history of fever (aPRR 2.23; CI 1.18–4.24) and seizures (aPRR 1.12; CI 1.09–1.16). Confirmed malaria was significantly lower among girls than boys (aPRR 0.92; CI 0.91–0.93), HIV infected children (aPRR 0.60 CI 0.52–0.71), and children with diarrhea (aPRR 0.76; CI 0.65–0.90). The overall prevalence of anemia (Hgb<10 g/dl) was 56.3% and severe anemia (Hgb<6 g/dL) was 17.8%. Among children with severe anemia 76.8% had malaria parasitemia, of whom 93.1% received blood transfusion. Malaria associated mortality was 0.6%.

**Conclusion:**

There was a high prevalence of malaria parasitemia and anemia among inpatient children under five years. Malaria prevention is a priority in this population.

## Introduction

There are 300–500 million annual clinical cases and nearly one million deaths from malaria in children globally, 90% of which occur in sub-Saharan Africa [1], [2]. In Uganda, malaria is endemic in more than 95% of the country and is the leading cause of morbidity and mortality, accounting for 25–40% of all outpatient visits, 20% of hospital admissions, and 9–14% of inpatient deaths [3], [4], [5]. *Plasmodium falciparum*, the predominate species, accounts for 99% of all malaria infections in Uganda [2], [4]. Approximately 70% of the country experiences very high transmission levels, 20% medium to high and 10% low transmission defined as >100, 10–100 and <10 infective bites per person per year, respectively [3]. Children under five years, HIV-positive persons and pregnant women bear the greatest burden of the disease [4].

Scale up of interventions to prevent and/or treat malaria such as use of artemisinin-based combination therapy (ACT), distribution of insecticide treated bed nets (ITNs), intermittent preventive treatment of pregnant women and indoor residual spraying (IRS) have reduced malaria prevalence in some endemic settings [4], [6], [7], [8] but this is not uniform across regions [9], [10], [11]. *Plasmodium falciparum* resistance to artemisinins and mosquito resistance to pyrethroids in ITNs have been reported and potentially threaten malaria control [2]. Improved monitoring of the effectiveness of current control strategies and development of new technologies such as malaria vaccines are critical to the continued success of malaria control [12], [13], [14], [15]. The testing of new malaria control tools and the design of future malaria control programs are contingent on understanding the current epidemiology of malaria and disease burden.

In many endemic settings efforts to evaluate the impact of interventions on malaria infections has mainly focused on community-based surveys [2], [4], [6]. There is, however, a paucity of high-quality facility-based data on in-patient malaria infections and deaths, and it cannot be assumed that the reduction of malaria at the community level translates into reduced hospital admissions for malaria. In Uganda, inpatient data is not systematically collected or is inadequate and incomplete due to a weak health management information system (HMIS) [4]. We assessed the prevalence, presentation, and treatment outcomes of malaria and anemia among children under five years hospitalized to two health facilities in rural Rakai district, South Western Uganda.

## Materials and Methods

### Ethics statement

The study was approved by Uganda Virus Research Institute-Science and Ethics Committee (UVRI-SEC), the Uganda National Council of Science and Technology (UNCST) and the Indiana University Institutional Review Board. Parents or guardians of children enrolled in the study provided written informed consent for their children before the children enrolled into the study. A written parental/guardian consent was also obtained for children who were tested for HIV.

### Study design and settings

This was a health facility-based observational study designed to estimate the prevalence, presentation and treatment outcomes of confirmed malaria cases in children under five years admitted at two health facilities in Rakai, Southwestern Uganda. Rakai district is on a plateau at an altitude ranging between 2500 and 3000 feet and has two rainy seasons. Malaria is meso- to holo-endemic with year round transmission and increased intensity during the rainy seasons or in communities adjacent to lakes and other mosquito breeding sites [16]. Kalisizo hospital (KH) is a Ministry of Health (MoH) facility providing outpatient and inpatient services. It is the main hospital for Kyotera, the largest town in Rakai district, and serves as the referral hospital for lower level health facilities within this sub-district with a catchment population of approximately 70,000 people. The hospital has a pediatric ward of about 50 beds and on-site laboratory facilities for basic microscopy. The hospital provides blood transfusion services but these services are not consistently available. The other facility was Bikira Health Center III (BHC), a private not for profit (PNFP) mission facility that provides outpatient and inpatient services located approximately 3.5 km from Kyotera town. BHC has a catchment population of approximately 30,000 people, with on-site laboratory facilities for basic microscopy and a 40 bed pediatric ward. This facility provides blood transfusion services and serves as a referral site for blood transfusion within Rakai district.

### Study Population

Children were eligible for this study if they were under five years of age and admitted to the inpatient wards at the two participating facilities. We sought written parental or guardian consent to enroll children within 24 hours of admission, but some children under five years were not enrolled. An effort was made to only enroll first admissions, but some repeat admissions or referrals between the two facilities may have occurred.

### Study procedures

In this study, all clinical and laboratory procedures were routine clinical care procedures conducted following Uganda MoH clinical guidelines [17]. There were no additional interventions assigned to clients. Blood was collected using a heel prick in infants or finger prick in older children to prepare blood smears for malaria parasites (MPs), hemoglobin (Hgb) and HIV testing. Thick and thin blood smears were prepared for malaria microscopy using Field stain A and B. Hgb was estimated using Hemocue (HemoCue AB, Box 1204, SE-262 23 Ängelholm Sweden). HIV rapid testing was conducted using the Determine® and Statpak® tests run in parallel. If the initial rapid tests were discordant a third rapid test (Unigold®) was run as a tie-breaker. For children over the age of 18-months, concordant positive results on the screen or Unigold® was positive results were considered as HIV infection, and concordant negative results were considered uninfected. For children less than 18-months of age, two concordant positive rapid tests were considered evidence of HIV-exposure. All HIV exposed children were referred for HIV DNA/PCR performed by the MoH Central public health laboratories (CPHL) in Kampala. HIV infected children were referred for HIV care and treatment.

Two laboratory technicians from each facility received a 2-week intensive training in microscopic examination of blood smears for MPs at the Malaria Diagnostic Center in Kisumu, Kenya [18]. The study provided a microscope, slides, and reagents. The quality of malaria microscopy slides and Hgb estimation was supported by external quality control; every 3 months, a 10% random sub-sample of slides was sent for re-reading by senior laboratory technologists at the RHSP laboratory. The Hemocue was calibrated daily by the Rakai Health Sciences Program (RHSP) laboratory and proper use was periodically assessed.

The facilities treated malaria according to Uganda MoH clinical guidelines [17]. The main drug used for severe malaria was intravenous quinine and artimesinin-lumenfatrine combination therapy (ACT) was the first line treatment for uncomplicated malaria.

### Data collection and management

Trained hospital staff collected enrollment and follow-up data using standardized case report forms (CRFs). Information included the child's gender, date of birth, date of admission and location; presenting symptoms and duration; temperature, weight, mid-upper arm circumference, level of consciousness, pulse, respiration rates, treatment (including blood transfusion) and treatment outcomes (discharge, referral, death or unknown). MPs results were abstracted from the laboratory request forms and Hgb and HIV tests were recorded directly into the CRFs. CRFs were entered into an electronic database in Visual FoxPro by trained RHSP data entry clerks.

### Data analysis

Malaria parasitemia (MP), was defined as the presence of any asexual (or other forms) of malaria parasites on a thick blood smear. Febrile illness was defined as an axillary temperature of ≥38°C. Anemia was defined as Hgb concentration <10 g/dl, and categorized as severe (Hgb<6 g/dl), moderate (Hgb 6–7.9 g/dl) and mild (Hgb 8–9.9 g/dl). Severe anemia associated with malaria was defined as Hgb<6 g/dl and a positive smear. According to the Uganda MoH clinical guidelines, blood transfusion is indicated when the Hgb concentration is below 6 g/dl [17]. HIV infection was defined as outlined above.

We estimated the prevalence of malaria parasite positivity among children under five years by gender, age, presenting symptoms, mid upper arm circumference (MUAC) and HIV-1 status. The association between risk factors, presenting symptoms and MP was assessed using generalized linear models with binomial log link regression to estimate the prevalence risk ratios (PRR) and 95% confidence intervals (CIs) controlling for age, gender, MUAC, HIV status and adjusting for clustering of children at health facility level. Anemia was excluded from this analysis and analyzed independently. In this setting where malaria is endemic, anemia is more likely to be a consequence rather than a risk factor of malaria. Student's t-tests were used to compare mean ages of children with and without malaria parasites. Treatment outcomes and factors associated with treatment outcomes were estimated using bivariate and multivariable analyses. All statistical analyses were conducted using STATA software package version 12 (College Station, Texas, USA). P values were double sided and considered significant when <0.05.

## Results

A total of 3727 children under five years were hospitalized at the two health facilities between May 18^th^, 2011 and May 31^st^, 2012, of whom, 2507 (67.3%) were enrolled into the study. However, 36 (1.4%) observations were excluded because the children either had duplicate identification numbers, missing age or failed to meet enrollment criteria (i.e. age ≥5 years), leaving an analysis sample of 2471. Greater than 95% of clients who were approached enrolled in the study.

Table 1 outlines the characteristics of the analysis sample. The majority of enrolled children were female (53.5%), admitted to KH (67.6%) and one year of age or younger (64.6%). The most commonly reported symptoms at presentation were fever (96.2%), cough (61.7%), vomiting (44.2%), diarrhea (20.8%) and seizures (16%). At admission, a clinical diagnosis of malaria (without laboratory evidence) was made in 94.5% of children. The mean age at admission was 1.3 years, (CI 1.25–1.35) and children with a laboratory diagnosis of malaria (1.4 years, CI 1.3–1.5) were older than those without malaria (1.2 years, CI 1.1–1.2; p<0.001).

**Table 1:** Characteristics of children at hospitalization in Bikira and Kalisizo health centers.

Characteristics		Total n (%)	Kalisizo Hospital n (%)	Bikira Health Center n (%)
**Total n (%)**		**2471 (100)**	**1671 (67.7)**	**800 (32.3)**
**Gender**	Male	1148 (46.5)	789 (47.2)	359 (44.9)
	Female	1323 (53.5)	882 (52.8)	441 (55.1)
**Age in completed years**	0	746 (30.2)	485 (29.0)	261 (32.6)
	1	849 (34.4)	553 (33.1)	296 (37.0)
	2	428 (17.3)	300 (18.0)	128 (16)
	3	276 (11.2)	188 (11.3)	88 (11.0)
	4	172 (7.0)	145 (8.7)	27 (3.4)
**Presenting symptoms at admission**				
	Fever	2376 (96.2)	1585 (94.9)	791 (98.9)
	Cough	1525 (61.7)	918 (54.9)	607 (75.9)
	Vomiting	1091 (44.2)	674 (40.3)	417 (52.1)
	Seizure	395 (16.0)	208 (12.4)	187 (23.4)
	Diarrhea	514 (20.8)	358 (21.4)	156 (19.5)
**Febrile illness (axillary tempt ≥38°C)**				
	Not Febrile	1243 (50.3)	810 (48.5)	433 (54.1)
	Febrile	1228 (49.7)	861 (51.5)	367 (45.9)
**Haemoglobin (Hgb) level (g/dl)**				
Severe Anemia	<6	439 (17.8)	86 (5.1)	353 (44.1)
Moderate Anemia	6.0–7.9	321 (13.0)	166 (9.9)	155 (19.4)
Mild Anemia	8.0–9.9	631 (25.5)	499 (29.9)	132 (16.5)
Normal Hgb	≥10	1080 (43.7)	920 (55.1)	160 (20)
**HIV Status**	HIV negative	2120 (85.8)	1361 (81.4)	759 (94.9)
	HIV positive	65 (2.6)	48 (2.9)	17 (2.1)
	Unknown	286 (11.6)	262 (15.7)	24 (3.0)
**Blood transfusion**	Yes	503 (20.4)	80 (4.8)	423 (52.9)
	No	1966 (79.6)	1590 (95.2)	376 (47.0)
**Diagnosis at admission**	Other Diseases	136 (5.5)	96 (5.7)	40 (5.0)
	Clinical malaria	2335 (94.5)	1575 (94.3)	760 (95.0)
**Malaria parasitemia**	Yes	1350 (54.6)	748 (44.8)	602 (75.2)
	No	1121 (45.5)	923 (55.2)	198 (24.8)
**Treatment outcome**	Discharged	2375 (96.1)	1590 (95.2)	785 (98.1)
	Dead	25 (1.0)	19 (1.1)	6 (0.8)
	Referred	39 (1.6)	32 (1.9)	7 (0.9)
	Unknown	32 (1.3)	30 (1.8)	2 (0.3)
**Duration of Hospitalization in days**				
	<2	237 (9.6)	202 (12.1)	35 (4.4)
	2 Days	1342 (54.3)	996 (59.6)	346 (43.3)
	≥3 Days	891 (36.1)	473 (28.3)	418 (52.3)

The overall prevalence of confirmed malaria parasitemia was 54.6% (Table 2). Parasitemia was significantly associated with a history of fever (aPRR 2.23; CI 1.18–4.24) and seizures (aPRR 1.12; CI 1.09–1.16). The risk of confirmed malaria was lower among female than male children (aPRR 0.92; CI 0.91–0.93) and among children with diarrhea than children without (aPRR 0.76; CI 0.65–0.90). Although the risk of confirmed malaria increased with age from 1 to 3 years, this was not statistically significant. The proportion of children with confirmed malaria parasitemia was significantly higher at BHC (75.2%) than at KH (44.8%; p<0.001). Comparing to the normal children, the prevalence of malaria was significantly lower among the severely malnourished aPRR 0.85; (CI 0.75–0.96), but not moderately malnourished children, aPRR 1.03; (CI: 0.90–1.18).

**Table 2:** Prevalence and PRRs^1^ of confirmed malaria among hospitalized children at Bikira and Kalisizo health centers (May/2011–May/2012).

Factors		No. Of Cases with MPs^2^/Total	Prevalence (%)	Unadjusted PRRs (95% CI^3^)	Adjusted PRRs (95% CI)
**Total**		1350/2471	54.6		
**Febrile illness (axillary tempt ≥38°C)**					
	Not Febrile	613/1243	49.3	1.00	
	Febrile	737/1228	60.0	1.22 (0.92–1.61)	
**Gender**					
	Male	658/1148	57.3	1.00	1.00
	Female	692/1323	52.3	0.91(0.91–0.92)	0.92 (0.91–0.93)
**Age in completed years**					
	0	343/746	46.0	1.00	1.00
	1	470/849	55.4	1.20 (0.76–1.89)	1.18 (0.76–1.83)
	2	270/428	63.1	1.37 (0.80–2.36)	1.30 (0.79–2.11)
	3	181/276	65.6	1.42 (0.77–2.63)	1.33 (0.77–2.31)
	4	86/172	50.0	1.09 (0.50–2.34)	1.00 (0.50–1.98)
**Presentation at admission**					
Fever	No	21/95	22.1	1.00	1.00
	Yes	1329/2376	55.9	2.53(1.32–4.86)	2.23(1.18–4.24)
Cough	No	533/946	56.3	1.00	
	Yes	817/1525	53.6	0.95(0.68–1.34)	
Vomiting	No	769/1380	55.7	1.00	
	Yes	581/1091	53.3	0.96(0.87–1.05)	
Seizures	No	1078/2074	52.0	1.00	1.00
	Yes	272/395	68.9	1.28(1.12–1.46)	1.12(1.09–1.16)
Diarrhea	No	1140/1957	58.3	1.00	1.00
	Yes	210/514	40.9	0.70(0.53–0.93)	0.76(0.65–0.90)
**HIV infection**					
	No	1214/2120	57.3	1.00	1.00
	Yes	22/65	33.8	0.59 (0.55–0.63)	0.60 (0.52–0.71)
	Result unknown	114/286	39.9	0.70 (0.55–0.89)	0.72 (0.58–0.88)
**Mid Upper Arm Circumference** 4 **in cm**					
	Green (≥13)	1194/2104	56.8	1.00	
	Orange (12- <13)	54/101	53.5	0.94 (0.67–1.33)	
	Red (<12)	21/50	42.0	0.74 (0.49–1.12)	

^1^ Prevalence risk ratios.

^2^ Malaria Parasites.

^3^ Confidence interval.

≥6 and <60 months; green-well nourished; orange-at risk of malnourishment; red-severely malnourished.^4^ For children

There were 77 HIV exposed children of whom 84.4% (n = 65) were confirmed HIV infected. The overall prevalence of HIV infection among hospitalized children under five years was 2.6% of whom 38.5% (n = 25) were confirmed to be under HIV care. HIV infected children were less likely to be hospitalized and diagnosed with malaria than HIV uninfected children (aPRR 0.60 (CI 0.52–0.71). The majority of the HIV infected children (61.5%) were less than 2 years of age.

At admission, the mean hemoglobin concentration of the study population was 9.0 g/dl (CI 8.9–9.1). The overall prevalence of anemia was 56.3% (17.8% severe, 13% moderate and 25.5% mild anemia). The mean Hgb was significantly lower in children with parasitemia as compared to children without (8.3 g/dl versus 10.0 g/dl, p = 0.001), and the prevalence of confirmed malaria was higher among children with lower Hgb (*X* ^2^ for trend p<0.001).

Of the 503 (20.4%) children who received blood transfusion, 79.5% had severe anemia. Among the 439 children with severe anemia, 76.8% had parasitemia and 93.1% received a blood transfusion. BHC transfused significantly more children (52.9%) than KH (4.8%); p<0.0001; (Table 1). The prevalence of anemia was 69.8% among severely and 64.9% among moderately malnourished children, compared with 55.3% among normally nourished children; (*X* ^2^ for trend p = 0.004). Children with either anemia or severe anemia were at no increased risk of death when compared to other children, (p = 0.99 and p = 0.46, respectively).

Overall, treatment outcomes were available for 2439 children. Of these, 2375 (97.4%) were discharged, 25 (1.0%) died and 39 (1.6%) were referred. Children with confirmed malaria had significantly lower case fatality (0.6%) compared to children without malaria (1.6%); p = 0.02. Mortality among enrolled children was 1.0% compared with 1.6% in non-enrolled children; p = 0.051.

## Discussion

This study documented a high prevalence of malaria parasitemia and anemia, but a low malaria case fatality among hospitalized children in Rakai district, Southwestern Uganda. Over half of the admissions in children under five years were diagnosed with malaria. Despite scale up of malaria prevention and treatment [6], [7], [8], malaria remains a major cause of hospitalization among children under five years. A similar study in Western Kenya reported a malaria prevalence of 82.5% in hospitalized children [19]. Other recent studies however, have reported declining prevalence of malaria attributed to interventions to prevent and/or treat malaria [6], [20], [21].

Nearly all (94.5%) of the inpatient children under five years were presumptively diagnosed with clinical malaria, yet the lab confirmation showed only 56.4% of these children had malaria parasitemia, suggesting substantial over diagnosis based on clinical presentation. This is consistent with other studies [22], [23] and suggests over diagnosis and inappropriate treatment of malaria [24]. Severely ill children who usually die within a few hours of admission especially before investigations are completed however may bias these findings and may also explain the higher mortality among the non-enrolled children.

HIV infected children had a lower prevalence of malaria than uninfected children, possibly because 38.5% of HIV infected children were on co-trimoxazole prophylaxis which has been shown to reduce liver stages of P. falciparum [25] and were provided with ITN as part of the basic HIV care package. However, HIV infected children are also at higher risk of presenting with infections other than malaria due to their immune-compromised status.

Although both malaria-associated fatality and all cause case fatality were low in our study population, children who were diagnosed with malaria were less at risk of death than those who actually did not have malaria. This difference in case fatality is consistent with a study in a Ugandan teaching hospital which documented a 1.5 times higher risk of mortality among smear negative children and a more than 3-fold increased risk of mortality amongst children who were presumptively diagnosed with malaria when compared to those with a positive malaria smear [26]. Children with a confirmed diagnosis of malaria are likely to receive prompt and proper treatment while those without malaria may be misdiagnosed and inappropriately treated with malaria drugs. Our study documented treatment details, but they are not included in this analysis.

Anemia was diagnosed in over half of the children, and was 68.7% in children with MPs consistent with other studies from similar settings [19]. Given that malarial anemia is multi-factorial, involving hemolysis and accelerated clearance of RBCs that continues for several months after malaria clearance [27], [28], many children are likely to be anemic at the time of their next malaria infection. Unlike previous findings [19], we did not find that anemia was a risk factor for death, probably because blood transfusion was administered to nearly all severely anemic children.

Our study had several limitations. Data collection was done by health workers from the health facilities who were inexperienced in research and placed priority on clinical care. However, all staff were trained on the protocol and good clinical practice. The RHSP quality control team audited all CRFs to ensure that any errors were corrected by study nurses (usually while patients were still hospitalized on the wards) before data entry. Although the study was designed to enroll all children, it was not possible in practice; some parents declined to participate, or their children were too sick to obtain parental consent, while others were admitted late at night and missed investigations such a blood smear, Hgb, etc prior to treatment. The study also failed to enroll some children who died before they had an opportunity to be enrolled, as indicated by the higher but not statistically significant mortality among non-study admissions. A concerted effort was made to only enroll first admissions at each institution, but some repeat admissions or referrals between the two facilities may have occurred.

Unlike other retrospective studies using routinely reported clinical records [19], [26]; our study prospectively documented the burden of malaria with external quality assurance for diagnosis of malaria and anemia.

## Conclusions

Malaria parasitemia and anemia were the two most common diagnoses among hospitalized children under five years in this rural Ugandan population. Although malaria case fatality was low, malaria prevention and access to early, effective treatment needs to be improved and consistently monitored to reduce the burden of life-threatening malaria and to effectively manage other non-malarial illnesses.

## Supporting Information

Study Approval S1 (ZIP)

Protocol S1 (ZIP)
