# Supplementary material for: High Prevalence of Malaria Parasitemia and Anemia among Hospitalized Children in Rakai, Uganda
Source: PLoS One. 2013 Dec 17;8(12):e82455. doi: 10.1371/journal.pone.0082455 (PMC3866122; doi:10.1371/journal.pone.0082455)
Supplement: Protocol S1 — (ZIP) [file pone.0082455.s002.zip › Protocol S1.pdf]

# RAKAI HEALTH FACILITY-BASED MALARIA OBSERVATIONAL STUDY

## A Study of

## The International Epidemiological Databases to Evaluate AIDS (IeDEA)

## And

## The Rakai Health Sciences Program (RHSP)


**Principal Investigators:** **Noah Kiwanuka M.D., PHD,**
**Yiannoutsos Constantin, PHD**

**Co- Investigators:**
**1) Kara Wools-Kaloustian M.D., M.S.**
**2) Wendy Prudhomme O'Meara, PhD**
**3) Valerian Kiggundu, MD, MPH**

**List of abbreviations**

| | |
|---|---|
| EIA | Enzyme-linked Immunosorbent Assay |
| IMCI | Integrated Management of Childhood Illnesses |
| HMIS | Health Management Information System |
| NIH | National Institutes of Health |
| OPD | Outpatients department |
| PNFP | Private-Not-For-Profit |
| RCCS | Rakai Community Cohort Study |
| RHSP | Rakai Health Sciences Program |
| UVRI | Uganda Virus Research Institute |

# 1. BACKGROUND AND RATIONALE

Malaria causes about 300-500 million clinical cases and nearly one million deaths in children globally each year, 90% of which occur in sub-Saharan Africa [1]. In Uganda, malaria is endemic in 95% of the country and is the leading cause of morbidity and mortality, accounting for 25-40% of all outpatient visits at health facilities, 20% of hospital admissions, and 9-14% of inpatient deaths (Uganda Ministry of Health, unpublished). Children under five years and pregnant women bear the greatest burden of the disease. Mitigating the terrible burden of malaria will require an effective anti-malaria vaccine [2,3] and efforts toward this goal are already underway with a target of licensing a vaccine which protects against 50% of severe disease episodes by 2015 [4]. Malaria vaccine trials will be conducted over the coming years but the design of these trials will be contingent on understanding the epidemiology of malaria and disease burden in different epidemic settings. In addition, evaluating the efficacy of an anti-malarial vaccine and interpreting the results of a malaria vaccine trial require an understanding of the epidemiological context in both clinical and community settings– including transmission intensity, infection prevalence, and disease burden [5]. Malaria transmission intensity is heterogeneous [6,7] and extrapolating the results of a trial to other endemic settings requires an understanding of the relationship between the epidemiological context and the resulting burden of disease [8-12].

The Rakai Community Cohort Study (RCCS), a population-based cohort in Rakai district south western Uganda that has been under surveillance since 1994, is a potential site for conducting malaria vaccine efficacy studies. Rakai district is situated on the plateau with an altitude varying between ~2500 to over 3000 feet, and two rainy seasons. Malaria is meso- to holoendemic, with year round transmission and increased intensity during the rainy season or in communities adjacent to lakes and other mosquito breeding sites [13]. Currently, data on malaria infections in Rakai district are limited and scanty

We propose to conduct a facility-based malaria observational study in Kalisizo and Bikira health center III in Rakai district, Uganda, to determine the proportion of patients with febrile illness (body temperature >=38$^0$c) that have malaria parasites on microscopy, and the proportions with severe malaria among children <5 years admitted to pediatric wards and malaria treatment outcomes among these children by HIV-1 serostatus.

The results of this study will improve the diagnosis and treatment of malaria cases in facilities and the management of non-malaria febrile illness (body temperature >=38$^0$c) in those who might have been diagnosed with malaria based on clinical symptoms in the absence of accurate testing. In addition, this study's results are expected to complement results from a community-based household observational study that will be conducted in communities of the Rakai Health Sciences Program cohort. Results from both studies will enhance the investigators' understanding of the epidemiology of pediatric and adult malaria infection in Rakai district in preparation for future malaria vaccine trials.

## 2. **STUDY OBJECTIVES**

### 2.1 **Objective 1**

To strengthen laboratory capacity and technical expertise at Kalisizo hospital and Bikira health center to provide routine parasitological confirmation of suspected malaria by microscopy and maintain a sensitivity and specificity of routine microscopy >90% as compared to reference microscopy.

### 2.2 **Objective 2**

To estimate the proportion of febrile illnesses (body temperature >=38$^0$c) with malaria parasites on a blood smear among all patients presenting to outpatient clinic at Kalisizo hospital

### 2.3 **Objective 3**

To estimate the prevalence, presentation, and treatment outcomes of severe malaria amongst HIV-infected and uninfected children aged <5 completed years admitted to Kalisizo and Bikira hospitals in Rakai district, Uganda.

### 2.4 **Objective 4**

To evaluate the feasibility of indentifying  inpatient children with severe malaria that come from households within RCCS and linking them to RCCS database in order to assess the relationship between household and socio-demographic factors and the burden of malaria, and the progression to severe disease and death in HIV infected and uninfected children. *This objective will only be undertaken if additional funding is received from NIH for this objective.*

## 3. **METHODS**

### 3.1 **STUDY SETTINGS**

The proposed study will be a descriptive cross sectional study conducted for 12 months at Kalisizo hospital and Bikira Heath Center III in Rakai district, Uganda.  The catchment area of these health facilities encompasses two clusters under surveillance by the Rakai Health Sciences Program (RHSP) cohort observational study.  Although RHSP has a memorandum of Understanding with Ministry of Health which supervises both health units and RHSP has worked and still works with health facilities in Rakai district, support letters from these particular facilities for this study will be sought and obtained before commencement of the study activities. Since the study will not be providing malaria treatment, the facilities will continue to treat malaria as they normally do.

Kalisizo hospital

Kalisizo hospital is a public health facility that provides both outpatient and inpatient services. It is the main hospital for Kyotera health sub-district and is located in the second largest town in the district, population 70 000, and is 1km from the RHSP field headquarters.  The hospital has

pediatric ward of about 50 beds, a maternity ward of 25 beds, a general medical-surgical ward of ~60 beds and on-site laboratory facilities for performing basic microscopy and other tests.

<u>Bikira Health Center</u>
Bikira health center III is a private not for profit (PNFP) missionary facility which provides both outpatient and inpatient services. It is located in a small village town in the district, population ~30,000, and is 15 km from the RHSP headquarters. The hospital has on-site laboratory facilities for performing basic microscopy. It has a pediatric ward of ~ 40 beds, a maternity ward of 15 beds, a general medical ward of ~30 beds.

<u>The Rakai Health Sciences Program (RHSP)</u>
RHSP in rural southwestern Uganda represents one of the largest and longest-running population–based research programs in sub-Saharan Africa. RHSP is a collaboration between the Uganda Virus Research Institute (UVRI), scientists at Makerere and Johns Hopkins Universities and intramural NIH via an International Center of Excellence in Research (ICER) award.

Since 1994, RHSP has conducted annual surveillance in a population of 12-14,000 persons under the Rakai Community Cohort Study (RCCS). RCCS is an on-going population–based, observational open-cohort study designed to monitor HIV-1 prevalence and incidence, and risk factors in Rakai district, Uganda. At the beginning of every survey round, a household enumeration census data are collected and updates made on number of persons in a household, their ages, relation to head, migration, and household structure and dwelling characteristics, and indices of socio-economic status at household level e.g., possessions (bicycle, motorcycle, car, land), source of water, availability of electricity etc. Eligible participants who provide written informed consent are enrolled as individuals, interviewed and followed up at home every 12-15 months and biological specimens for HIV and STI testing are collected. Free voluntary HIV counseling and testing as well as treatment for general ailments and STI symptoms are provided. Since June 2004, via President's Emergency Plan for AIDS Relief (PEPFAR) support, RHSP provides HIV care for over 5,000 HIV-infected persons, which includes cotrimoxazole prophylaxis, provision of bed nets, and antiretroviral drugs which have substantially reduced disease burden in the district.

### 3.2 **STUDY DESIGN**
The proposed study is a 12 months descriptive cross-sectional health facility-based study that will be conducted in Kalisizo hospital and Bikira health center III, in Rakai district, Uganda. It will involve retrospective review of OPD and lab records and enrolment and observation of children <5 years admitted to the aforementioned health facilities.

### 3.3 **STUDY POPULATION**
For objective 2, all OPD and lab records for the 12 months study period will be reviewed at monthly to determine the proportion of patients with febrile illness (body temperature $>=38^{0}$c) that have positive malaria smear results. However, for objective 3 and 4 (if funded), we expect

to enroll and observe about ~3000 children aged <5 years admitted to pediatric wards in Kalisizo hospital and Bikira health center III.

### 3.4 **PROCEDURES**

3.4.1 Strengthening laboratory capacity

To achieve the objective of strengthening laboratory capacity and technical expertise in the Kalisizo hospital and Bikira health center to provide routine parasitological confirmation of suspected malaria by microscopy, the study will provide a microscope, slides, and blood smear reagents to Kalisizo hospital and Bikira health center, and refresher training in microscopic examination of blood smears for malaria parasites and ongoing quality control of malaria diagnostic services in each laboratory. Two technicians from each facility will be trained at the malaria training center lab in Kisumu, Kenya. At each lab, patients with febrile illnesses (body temperature >=$38^0$c) sent by clinicians for malaria blood smears will be received by the technicians and their socio-demographics and identification numbers will be recorded in the lab book. Finger prick or heel prick blood will be collected onto a slide, air dried and stained using field stain. Presence of malaria parasites will be detected under a microscopy using high power magnification. Results will be reported on to the lab request form as well as in the lab book. For quality control purposes, every 3 months a 10% random sub-sample of slides from Kalisizo hospital and Bikira HC laboratories will be selected and sent for re-reading by senior lab technologists at RHSP lab. An abbreviated re-fresher training will be provide to two RHSP senior lab technologist that will be re-reading the slides.

3.4.2 Estimating the proportion of febrile illnesses with positive malaria blood smears

To achieve this objective, the study will, in addition to strengthening lab capacity for routine parasitological confirmation of suspected malaria by microscopy, provide re-fresher training to health care providers on case management of febrile illnesses with emphasis on IMCI principles of febrile case management. Training will be based on WHO and MoH standard guidelines for diagnosis and treatment of malaria including the interpretation and use of blood smear test. De-identified data will be abstracted from OPD and lab record books from Kalisizo hospital to determine the number of patients who presented to OPD with febrile illness (body temperature >=$38^0$c), number of patients with febrile illness (body temperature >=$38^0$c) who sent to the lab for malaria testing, and the number of febrile illness (body temperature >=$38^0$c) patients with positive malaria smear results. This will be done monthly by a study nurse who will send the abstracted data to the Data Manager at RHSP through the field coordinator. The population for this objective is all patients presenting to OPD with a febrile illness.

3.4.3 Prevalence and treatment outcomes of severe malaria in admitted children

To estimate the prevalence, presentation, and treatment outcomes of malaria amongst HIV-infected and uninfected in-patient children, all children aged ≤5 years admitted to Kalisizo hospital and Bikira health center will be approached for enrollment into the study. The will be study explained to parents/caregivers of eligible patients and if they (parents/caregivers) agree, an informed consent will be administered. Blood will be collected for malaria microscopy (4 drops), hemoglobin level (2 drops), and for HIV rapid testing (4 drops). Presence or absence of

malaria parasites and parasite density will be determined by microscopy. Hemoglobin will be determined by Sahl's method which is routinely used in non-research settings in Uganda. A study standardized inpatient data collection form (see appendix) will be used to collect data on the following:
1) Patients particulars – names, gender, data of birth, date of admission, and location.
2) Presenting symptoms and duration of each symptom
3) Clinical examination findings – temperature, weight, height/length, mid-arm circumference, heart rate, respiratory rate, general body exam, and level of consciousness.
4) Laboratory results, blood transfusion, treatment/medication, treatment outcome, and counseling.
This will involve all individuals because it is a clinical strengthening exercise. We will not be doing additional data collection on adults beyond what is done in the normal course of clinical care. We will request data from the registers maintained by the facilities but the data requested will be de-identified and unable to be linked to an individual patient hence no additional consent will be required.

For children whose parents/caregivers agree to HIV testing, voluntary HIV counseling will be provided to parents/caregivers and HIV results (with pre- and post-result counseling) will be returned to those who will express willingness to receive them. Parents/caregivers will be free to decide whether or not their child can be tested for HIV i.e., consent to participate in the study does not imply consent to HIV testing. Parents will have to right to decline their children's participation in some or all parts of the study. HIV rapid testing will be done according to the algorithm describe under laboratory methods (section 3.6). Children found HIV positive on rapid tests will be referred to RHSP HIV/ARV care program for further testing including HIV-DNA PCR and those infected will be treated according to World Health Organization guidelines for paediatric HIV/AIDS treatment and management. At RHSP, infants (less than 1 year) will be tested using HIV DNA PCR while other children will be tested using EIA tests.

A referral form will be completed in duplicate and one piece will be given to the caregiver who will be referred to the RHSP HIV/ARV satellite clinic nearest to their home. A log of referrals will be kept by the study and regular updates on those referred will be given to the RSHP HIV/ARV program office at RHSP headquarters in Kalisizo. The RHSP HIV/ARV care program is willing to take on children with HIV under its care. We will determine the proportion of children admitted for malaria among all pediatric admissions, and the proportion of hospitalized malaria, severe malarial anemia, and cerebral malaria by HIV status among those with malaria.

3.4.4 <u>Linkage of children with severe malaria to RCCS census database</u>
To assess the relationship between household and socio-demographic factors collected during RCCS census activities and morbidity/mortality associated with severe malaria, the study will identify patients that come from RCCS censused households and link them to RCCS census database so as to obtain their household level census data. The data will include age at the time of census, relation to head, migration, and household structure and dwelling characteristics, and indices of socio-economic status at household level e.g., possessions (bicycle, motorcycle, car, land), source of water, availability of electricity etc. Over the years, RHSP has developed

tool (linkage form) with a set of screening questions used to identify persons with a high index of suspicion for having been censused in RCCS in previous surveys. This linkage form (see appendix) will be administered to caregivers of admitted children as a screening tool. Forms of cases with a high likelihood of coming from previously censused RCCS households will be sent to RHSP data management center for electronic linkage. Verification and ascertainment field visits may be conducted to confirm the completeness/accuracy of linkage as is routinely done with linkages in the RCCS and other RHSP studies. Since all households in RCCS census database have GPS coordinates, that information plus other census data will be used create a spatial database of household socio-demographic characteristics including distance to a health care facility and indices of wealth. We will then determine the household and individual factors that contribute to increased risk of malaria hospitalization, severe malaria, and death. This objective will only be done if additional funds are provided by NIH.

3.5 **DATA MANAGEMENT AND ANALYSIS**

Study personnel will be trained on study goals, protocol and procedures, and data collection tools prior to study commencement. On a monthly basis, a study nurse will abstract de-identified data from OPD and lab record books on patients that presented with febrile illness (body temperature >=38$^0$c). Variables will include age, gender, village, temperature, referral to the lab for malaria microscopy, and results of malaria microscopy. In addition, data will be collected on the total number of patients that presented to OPD during the same time frame. For children <5 years admitted to pediatric wards, a study standardized inpatient data collection form (see appendix) will be used to collect data on the following:

1) Patients particulars – names, gender, data of birth, date of admission, and location.
2) Presenting symptoms and duration of each symptom
3) Clinical examination findings – temperature, weight, height/length, mid-arm circumference, heart rate, respiratory rate, general body exam, and level of consciousness.
4) Laboratory results, blood transfusion, treatment/medication and treatment outcome (discharge, referral, death and date of outcome)
5) Counseling for HIV, HIV testing and test results, and referral to RHSP HIV/ART care program.

Data will be collected by study nurses working on pediatric wards who will submit completed forms to the Data Manager through the Field Coordinator. Data will be entered in electronic format using entry screens in Visual FoxPro or MS Access. Analyses will involve estimation of

a) Point and period prevalence of febrile illnesses (body temperature >=38$^0$c) among patients reporting to OPD
b) Point and period prevalence of malaria parasite microscopy positivity among patients with febrile illness sent to the lab for testing. Comparisons will be done by gender and age. Multivariable logistic regression models with robust variance estimator will be used to determine the factors associated with malaria microscopy positivity. If the prevalence of the outcome turns out to be greater than 10% in which case odds ratios overestimate the true measure of association, we will use log-binomial regression models with either binomial link or log link to estimate prevalence risk ratios and their associated 95% confidence intervals.

c) Point and period prevalence of children under 5 years admitted with severe malaria and analyses will be stratified by HIV sero-status.
d) Proportions of treatment outcomes will be estimated for hospitalized malaria, severe malarial anemia, and cerebral malaria, and estimates will be stratified according to HIV-1 status for children under 5 years. Factors associated with treatment outcomes will be estimated using multivariable logistic regression models where the outcome prevalence is <=10% and log-binomial regression models where the outcome is >10%.

e) We will also estimate the proportion of admitted children <5 years that came from households within RCCS and through linkage to RCCS census database, we will determine the household and individual factors that contribute to increased risk of malaria hospitalization, severe malaria, and death using multivariable logistic regression models adjusting for correlation at household level. This analysis will only be done if additional funds are provided by NIH.

## 3.6 **LABORATORY METHODS**

Routine microscopy for malaria parasites will be done for OPD patients with febrile illness (body temperature >=38$^0$c) as requested by attending clinicians. Finger or heel prick (for infants) blood will be collected from patients presenting to lab with lab request forms by the lab technician. For in-patients, blood for malaria microscopy (thin and thick smear), hemoglobin levels, and HIV rapid tests will be collected using a heel prick in infants or finger prick in older children. Collection of finger /heel prick blood drops for malaria testing is a routine standard practice in health facilities in Uganda that have laboratory capacity to test for malaria parasites. Specimens will be collected on the ward by the study nurse and will be labeled with each patient's hospital identification number and taken to the lab for testing. The presence of malaria parasites, speciation, and parasite density will be reported on the lab request form and record in the lab log book alongside each patient's particulars as indicated by HMIS guidelines. Malaria microscopy slides will be kept for external quality control purposes and every 3 months, a 10% random sub-sample of slides from Kalisizo hospital and Bikira HC laboratories will be selected and sent for re-reading by senior lab technologists at RHSP lab. Hemoglobin will be determined by Sahl's method which is routinely used in non-research settings in Uganda. HIV rapid testing will be done using Determine® and Statpak® tests run in series; if both tests yield concordant test results, that result will be taken and reported but discordant results will be subjected to Unigold® as a tie-breaker.

The study will strengthen the laboratory capacity at Kalisizo hospital and Bikira health center to provide routine malaria parasitological confirmation by microscopy. This will be achieved through the provision of equipment (microscope, slides), supplies (blood smear reagents), refresher training in microscopic examination of malaria blood smears for malaria and ongoing quality control of malaria diagnostic services in each laboratory. Two technicians from each facility will be trained at the malaria training center lab in Kisumu, Kenya.

# 4. ETHICS/PROTECTION OF HUMAN SUBJECTS

### 4.1 Institutional Review Board

A copy of the protocol, informed consent forms, and other forms for data collection for the study will be submitted to the Science and Ethics Committee (SEC) of UVRI (the local IRB of record for all RHSP studies), the Uganda National Council for Science and Technology (UNCST) and to the IRB at University of Indiana, USA, for review and approval.

### 4.2 Informed Consent Process

Apart from determining the HIV status for children admitted to pediatric wards, administering an in-patient data collection form and linkage with RCCS census database, the rest of the procedures in this study are routine ones that are done normally in health facilities in Uganda. Such procedures include patients presenting at OPD, getting recorded in HMIS books, seeing a clinician, referral to the lab for malaria smear microcopy for patients with suspected malaria, admission of patients that clinicians deem sick enough for in-patients care, provision of medication, and referral or discharge depending on response to treatment. Data that will be abstracted from OPD and lab record books (to determine the number of patients who presented to OPD with febrile illness (body temperature >=38$^{0}$c), number of patients with febrile illness who sent to the lab for malaria testing, and the number of febrile illness patients with positive malaria smear results) are routinely collected as part of normal operations at these health centers. These data will be de-identified and aggregated for analysis. Thus, informed consent will be sought for enrolment of children <5 years admitted to pediatric wards. Written informed consent will be sought from the attending parent/caregiver. Administration of the consent form will be done by the study nurse who will discuss the study's purpose, procedures, risks, potential benefits, and the rights of participants with the parent/caregiver, to help him/her make educated/informed decisions about whether or not to allow his/her child to participate in the study. Ample time will be given to the parent/caregiver discuss any issues concerning the study and ask questions. Parents/caregivers will sign/thumb print and date the informed consent document prior to enrolment of their children in the study. The original signed informed consent form will be retained by the study and a copy will be provided to the participant's parent/caregiver.

### 4.3 Participant Confidentiality

The investigator will ensure that the participant's privacy and confidentiality is maintained. Participants will not be identified in any published reports of this study. All records will be kept confidential to the extent provided by the law. Personally identifiable information in electronic data management systems will be protected compliant with regulatory requirements and federal computing security requirements to preserve privacy and confidentiality.

**Appendix 1: Informed Consent document**

**CONSENT FORM for**
**RAKAI HOSPITAL -BASED MALARIA OBSERVATIONAL STUDY**

Hello, my name is ____________________________. I am working with researchers from Rakai Health Sciences Program (Uganda), Indiana University School of Medicine and George Washington University School of Public Health in the United States of America (USA). We are inviting your child to participate in a study on malaria in children, because your child has symptoms suggestive of malaria. Inviting your child to join this study does not mean that he/she has malaria parasites, but we will find out whether or not he/she has them when we do laboratory tests.

The study is being conducted by Dr. Noah Kiwanuka of Rakai Health Sciences Program working with investigators from Indiana University School of Medicine and George Washington University School of Public Health in the United States. It is funded by the National Institutes of Health in the United States.

**Study Purpose:** The purpose of this study is to determine how many children aged <5 years present to Kalisizo and Bikira hospitals in Rakai district, Uganda, with severe malaria and how they respond to treatment.

**Number of people taking part in the study**: If you agree to have your child participate, he/she will be one of up to 3,000 children participating in this study.

**Study Procedures:** If you agree to your child participating in this study, the following things will be done:

- You and your child will be asked a few questions about the child's symptoms and your child will be examined by a clinician who will look for signs of malaria such as fever, yellow eyes, or an enlarged spleen. The spleen is an organ located near the stomach that destroys worn-out blood cells and stores blood.
- We will ask for your permission to draw about 8-10 drops of blood for testing for malaria parasites, HIV, and hemoglobin (to see if your child has enough blood). One of the symptoms of insufficient blood is pale mucus membranes. Your child's participation in the study does not mean that's/he will be tested for HIV; if you allow your child be tested for HIV, you will have to provide additional permission for that. You will be given your child's HIV results only if you want them; if you do not want them, we will not give them to you. Blood will be collected by a prick on the finger (children aged 2 years or more) or a prick on a heel (for children less than 2 years).

- The information gathered by the clinician while asking questions and doing the exam, the results of the blood tests and the medications given to your child while in hospital will be recorded on a form that will be entered into a computer.
- You will be asked if you or your child has ever stayed in a household that is among those that the Rakai Health Sciences Program does research.

**Risks of participation and measures taken to minimize risk:**

Potential risks of participating in this study include the following:

1) There is the potential for confidential information on your child to be known by other people. We will minimize this risk by training all individuals working on this study in confidentiality procedures, and all patient paper data collected by the study will be kept in locked file cabinets when not in use by the hospital caring for the patient. Only persons authorized by the principal Investigator will have access to these cabinets. Computers will have password protection. We will keep your child's information confidential to the full extent allowed by the law. Your child's information may be disclosed if required by law. Your child's identity will be held in confidence in reports in which the study may be published.

2) Heel or finger prick may cause a small amount of pain and can be distressing to the child as well as having a very small potential for infection. However, this is a risk that would be encountered regardless of participation in this study, as blood testing would be done as standard of care for your child's illness.

**Benefits of participation:** The main benefit to your child will be access to testing for anemia, which is sometimes not routinely available at the hospital due to shortages in supplies. If your child is suspected of being HIV infected, s/he will be referred to Rakai Health Sciences Program for further testing and you will be the one to decide to take him/her there. If the tests done by Rakai Health Sciences Program show that your child is infected with HIV, s/he will be put on antiretroviral treatment. There may be future benefits to people in Rakai, if the study helps us understand the impact of malaria on children in the area.

**Alternatives to taking part in this study:** Instead of being in this study, your child can receive the standard health care provided at this facility for your child's illness.

**Costs:** There will be no additional cost to you for your child's participation in this study.

**Compensation for participation:** There is no monetary compensation for participation in the study.

**Compensation for Injury:** In the unlikely event of physical injury resulting from your child's participation in this study, necessary medical treatment will be provided by the study.

**Contacts for questions or problems:** If you have any questions, please ask, and I will do my best to answer them. If you have additional questions or if you need to discuss any other aspect of the service, you can contact: Dr. Noah Kiwanuka (0701444154), Dr. Godfrey Kigozi (0701444144), Dr. Kiggundu Valerian (0701444054) Medical Officers, Rakai Program, Kalisizo. You can also call Rakai Health Sciences Program office in Kalisizo (0772-405 861).

If you have any questions concerning your child's rights as a participant in this study, please contact the Chairman of Science and Ethics committee of UVRI (tel 0414-320385/6).

**Participation is voluntary**

Your child's participation in this study is entirely voluntary, you may refuse to have your child participate in the study or any part of the study and you can withdraw you child from the study at any time without losing access to health care and other benefits provided by the Rakai Health Sciences Program.

If there is any portion of this consent explanation sheet that you need clarified, ask the nurse before signing. You will receive a copy of this consent form.

**Consent to participate in the study (excluding HIV testing)**
The study has been explained to me and I agree to allow my son/daughter to participate in the study. I also have been informed that he/she also has the right to voluntarily refuse to participate in all or part of the service.

________________________________________ __________________
SIGNATURE OF PARENT OR GUARDIAN DATE
________________________________________ __________________

________________________________________ ______________
SIGNATURE OF PERSON ELICITING CONSENT DATE

**Consent for HIV testing**
I agree to allow my son/daughter to be tested for HIV, the virus that causes AIDS. I also have been informed that he/she also has the right to voluntarily refuse to participate in all or part of the service.

________________________________________ __________________
SIGNATURE OF PARENT OR GUARDIAN DATE
________________________________________ __________________

________________________________________ ______________
SIGNATURE OF PERSON ELICITING CONSENT DATE

**EKIWANDIIKO EKIRAGA OKUKKIRIZA OKUNYOONYERAZA N'OKWEKENENYA OMUSUJJA GW'ENSIRI MUMALWALIRO MU RAKAI.**

Nkulamusizza ssebo/nyabo, erinya lyange nze……………………………. Nkola n'abakola okunyoonyereza mu Rakai Health Sciences Program(Uganda),Indiana University School of Medicine ne George Washington University School of Public Health mu United States of America(USA). Tusaba omwana wo okwetaba mukunoonyereza okukwata ku musujja gw'ensiri mu baana,kubanga omwana wo alina obubonero obwefananyirizaako obw'omusujja gw'ensiri. Okusaba omwana wo okwetaba mu kunoonyereza kuno tekitegeeza nti alina obuwuka bw'omusujja gw'ensiri,naye tujja kukizuula oba abulina oba tabulina nga tukebera omusaay.

Okunoonyereza kukolebwa Dr.Noah Kiwanuka okuva mu Rakai Health Sciences Program nga akolaganira wamu n'abanoonyereza okuva mu Indiana University School of Medicine ne George Washington University School of Public Health mu United States. Abawomyemu omutwe beba National Institutes of Health mu United States

.

**EKIGENDERERWA KYOKUNOONYEREZA.**

Ekigendererwa kyokunoonyereza kuno kwe kumanya abaana bameka abatannaweza myaka etaano abajja mu ddwaliro lye Kalisizo ne Bikira mu Rakai District,Uganda,nga balina omusujja gw'ensiri ogwamaanyi n'engeri gyebayisibwamu nga funye obujjanjabi.

**OMUWENDO GW'ABANTU ABANETABA MU KUNOONYEREZA**.

Bw'okkiriza omwana wo okwetaba mu kunoonyereza ajja kuba omu kw'abo abaana enkumi satu (3,000) abanetaba mu kunoonyereza kuno.

**ENKOLA NGA BWENAABA**

Bw'okkiriza omwana wo okwetaba mu kunoonyereza kuno ebintu bino wamanga bye binakolebwa:

- Gwe n'omwana wo mujja kubuuzibwa ebibuuzo bitono ebikwata ku bubonero omwana bwanaaba nabwo era omwana wo ajja kukeberebwa omusawo okulaba oba alina obubonero bw'omusujja gw'ensiri okugeza nga okwokya/ebbugumu,amaaso agakyenvu,oba okuzimba akabengo. Akabengo kye kitundu ky'omubiri ekiriranye olubuto lwemmere ekisanyawo obutafaali bw'omusaayi obuba bwonoonese n'okutereka omusaayi

- Tujja kukusaba olukusa okujja ku mwana wo omusaayi amatondo nga 8-10 okukeberamu obuwuka bw'omusujja gw'ensiri,akawuka ka siriimu n'obungi bw'omusaayi mu mubiri(okulaba oba omwana wo alina omusaayi ogumala). Akamu kububonero obulaga nti omusaayi mutono bye bitundu by'omubiri nga wansi wemunye,olulimi okwerukirira. Omwana wo okwetaba mu kunonyoreza tekitegeeza nti ajja kukeberebwa akawuka ka siriimu,bw'okkiriza omwana wo okukeberebwa akawuka ka siriimu ojja kwongera okutuwa olukusa olulala. Ojja kumanyisibwa ebivudde mu kukebera omwana wo ebikwata ku kawuka ka siriimu singa onaaba obyagadde,singa onaaba tobyagadde tetujja kubikumanyisa. Akasaayi kajja kujjibwako nga

tufumita ku ngalo(mu baana ab'emyaka ebiri oba okusingawo) oba okufumita ku kakongovvule(mu baana aba tannaweza myaka ebiri).

- Ebinaaba bikubuuziddwa omusawo, ebinaava mu kwekebejja omwana wo ne binaava mu kukebera omusaayi wamu n'obujjanjabi omwana wo bw'anafuna ng'akyali mu ddwaliro bijja kuwandiikibwa ku lupapula olunakozesebwa okubiyingiza mu kyuma kikalimagezi(Computer).

- Ojja kubuuzibwa nti,gwe oba omwana wo waliwo eyali abaddeko munyumba abasawo ba Rakai Health Sciences Program mwebakola okunoonyereza.

**OBUZIBU OBUYINZA OKUBAWO OLWOKWETABA MU KUNOONYEREZA N'ENGRI GYEBUYINZA OKUKENDEZEBWAMU.**

Obuzibu obuyinza okubawo olwokwetaba mu kunonyereza kuno bwe buno:

1, Waliwo obuzibu bwebikubuuziddwa ebikwata ku mwana wo okutegerebwa abantu abalala. Tujja kukendeza kubuzibu buno nga tutendeka abasawo bonna abanakola okunoonyereza kuno mungeri zokukuumamu ebyama,era nebiwandiiko ebikwata ku balwadde mu kunoonyereza kuno bijja kukumibwa nga bisibiddwa muzikabada mubiseera lwebinaaba tebikozesebwa mu malwaliro abalwadde mwebafunira obujjanjabi. Abo bokka abakiriziddwa abakulira okunoonyereza bebanatukirira zikabada zino.Ebyuma bikalimagezi (Computers) bijja kuba nenamba ennekusifu(password protection) emanyiddwa abo bokka abagikozesa. Tujja kukuuma ebikwata ku mwana wo nga byakyama ng'amateeka bwegalagira. Ebikwata ku mwana wo biyinza okutegezebwako abantu abalala singa kinaaba kyetagisa okusinziira ku mateeka. Amannya g'omwana wo tegajja kubeera mu biwandiiko ebinafulumizibwa ebikwata ku kunoonyereza.

2, Okufumita akakongovvule oba engalo kuyinza okuleeta obulumi butono era kuyinza okweralikiriza omwana n'okumulwazaamu katono. Naye obuzibu buno bwandibaddewo nebweyandibadde teyetabye mu kunoonyereza kuno, kubanga yandibadde era ajjibwako omusaayi mu kujjanjabwa okwabulijjo okusinziira ku bulwadde bw'omwana.

**EMIGASO OBA BY'ONOGANYULWAMU**

Omugaso ogusinga eri omwana wo kwekukebera okulaba oba alina omusaayi ogumala,kino tekitera kukolebwa buli mulundi omuntu lwajja muddwaliro kubanga tewaberawo bikozesebwa.Singa omwana wo ateberezebwa okuba n'akawuka ka siriimu,ajja kuwerezebwa ku Rakai Health Sciences Program okwongera okukeberebwa era gwe ojja okusalawo okumutwalayo.Singa okukebera okunaakolebwa Rakai Health Sciences Program kulaga nti omwana wo alina akawuka ka siriim ajjakuwebwa eddagala erikendeeza obungi bw'akawuka ka siriimu mu musaayi(ART).Wayinza okubawo emigaso mubiseera oby'omumaaso eri abantu ba Rakai, singa okunoonyereza kunatuyamba okutegeera obulungi obuzibu bw'omusujja gw'ensiri mu baana bo mu kitundu kino.

**OKUSALWO OBUTETABA MU KUNOONYERZA.**

Nebwaba teyetabye mu kunoonyereza kuno,omwana wo asobola okufuna obujjanjabi bw'obulwadde bwalina okuva muddwaliro lino.

**OKUSASULA**
Tewali kwongera kusasula sente olw'omwana okwetaba mu kunoonyereza kino.

**OKUSASULIBWA OLW'OKWETABA MU KUNOONYERZA.**
Tewali sente zakusasulwa olw'okwetaba kunoonyereza.

**OKUSASULIBWA NG'ALWADDE**
Okufuna obulemu olw'okwetaba mu kunoonyereza kuno tekitera kubawo,naye singa kibaawo omwana wo ajjakufuna obujjanjabi bwonna obwetagisa.

**ABANTU BOYINZA OKUTUUKIRIRA NG'OLINA EBIBUUZO OBA EBIZIBU**.
Bw'oba olina ebibuuzo byonna ebikwata ku kunoonyereza kuno, bambi nkusaba obuuze era nange ndi mwetegefu okukola kyonna ekisoboka okubyanukula.Bw'oba olina ebibuuzo ebirala byonna oba nga wetaaga okukubaganyamu ebirowoozo ku bikwata ku kunoonyereza kuno, tuukirira abantu bano wamanga:
Dr Noah Kiwanuka (essimu 0701444154), Dr. Godfrey Kigozi (essimu 0701444144), Dr.Kiggundu Valerian (essimu0701444054) abasawo bebyobulamu mu Rakai Health Sciences Program ekalisizo.Osobola okukuba ku ya Rakai Health Sciences Program office e Kalisizo(essimu 0772405861).

Bw'oba olina ebibuuzo byonna ebikwata ku ddembe ly'omwana wo eyetabye mu kunoonyereza kuno tuukirira ssentebe w'akakiiko akalabirira ebya ssayansi ne ddembe ly'abantu abeetaba mu kunoonyereza asangibwa mu UVRI (essimu 0414-320385/6)

**OKWETABA MU KUNOONYEREZA OBA OKUKUVAAMU KWA KYEYAGALIRE.**
Omwana wo okwetaba mu kunoonyereza kuno kwa kyeyagalire, oli wa ddembe okugaana omwana wo okwetaba mu kunoonyereza kuno oba okugaanako ebitundu ebimu eby'okunoonyereza, era osobola okujja omwana wo mu kunoonyereza obudde bwonna notabaako kyofirwa ku bujjanjabi ne bintu ebirala Rakai Health Sciences Program byekola oba byegaba.

Bwe waberawo akatundu konna mu kiwandiiko kino kotategedde nga wetaaga okwongerwa okunyonyolwa,buuza omusawo nga tonasaako mukono/ekinkumu. Ojja kuweeba kkopi y'ekiwandiiko kino ekiraga okukkirizakwo.

**OKULAGA OKUKKIRIZA OKWETABA MU KUNOONYEREZA(NGA TEMULI KUKEBERA KAWUKA KA SIRIIMU)**
Ebikwata ku kunoonyereza kuno binyinyonyoddwa bulungi,era nzikiriza omwana wange okukwetabamu. Era ntegezeddwa nti omwana alina eddembe okugaana okwetaba mu kunoonyereza kuno oba okugaanako ebitundu ebimu eby'okunoonyereza.

---------------------------------------------------- -----------------
Omukono/ekinkuno ky’omuzadde oba amulabirira Ennaku Z’omwezi

---------------------------------------------------- -----------------
Omukono gw’omusawo Ennaku z’omwezi

**OKULAGA OKUKKIRIZA OKUKEBERA AKAWUKA KA SIRIIMU**

Nzikirizza omwana wange okumukebera akawuka ka siriimu,akaleeta obulwadde bwa mukenenya(AIDS), Era ntegezaddwa nti omwana alina eddembe okugaana okwetaba mu kunoonyereza kuno oba okugaanako ebitundu ebimu eby’okunoonyereza.

---------------------------------------------------- -----------------
Omukono/ekinkuno ky’omuzadde oba amulabirira Ennaku Z’omwezi

---------------------------------------------------- -----------------
Omukono gw’omusawo Ennaku z’omwezi

**Appendix 2: In-patient form**

<table>
<tr><td colspan="6">RAKAI<br>Pediatric In-patient<br>Malaria Encounter Form</td></tr>
<tr><td colspan="2">1. Name (3 names – given, middle, family):</td><td>2. Hospital Number:</td><td colspan="3">3. Study Number</td></tr>
<tr><td colspan="2">4. Date of Admission (dd/mm/yyyy):</td><td colspan="3">5. Date of Birth(dd/mm/yyyy):</td><td>6. Sex: ☐ M ☐ F</td></tr>
<tr><td colspan="3">7. Locator Information (Location, sub-location, village):</td><td colspan="3">8. Referred from:<br>Reason for referral ______________________________</td></tr>
<tr><td colspan="6">9. History: Has the patient had any of the following symptoms?</td></tr>
<tr><td colspan="6">a. Fever ☐ No ☐ Yes No. days _________<br>b. Cough ☐ No ☐ Yes No. days _________ Blood ☐ No ☐ Yes<br>c. Vomiting ☐ No ☐ Yes No. days _________ Blood ☐ No ☐ Yes<br>d. Seizures in the past 24 hours ☐ No ☐ Yes<br>e. other complaints: ______________________________</td></tr>
<tr><td colspan="6">10. Physical Exam:</td></tr>
<tr><td>Temperature</td><td>Weight</td><td>Height (Length)</td><td>Upper Arm Circumference</td><td>Heart Rate</td><td>Respiratory Rate</td></tr>
<tr><td></td><td></td><td></td><td></td><td></td><td></td></tr>
<tr><td colspan="4">General:<br>a. Trouble breathing, flaring or indrawing? ☐ No ☐ Yes<br>b. Oedema ☐ No ☐ Yes<br>c. Stiff Neck ☐ No ☐ Yes<br>d. Skin Infection ☐ No ☐ Yes<br>e. Skin Rash ☐ No ☐ Yes<br>f. Ear Discharg/pain ☐ No ☐ Yes</td><td colspan="2">Notes:</td></tr>
<tr><td colspan="4">Level of Consciousness: Can the child do the following?<br>a. Sit up ☐ No ☐ Yes<br>b. Suck or Swallow ☐ No ☐ Yes<br>c. Respond to stimuli ☐ No ☐ Yes</td><td colspan="2">Notes:</td></tr>
<tr><td colspan="6">11. Test Results:</td></tr>
<tr><td colspan="2">Hgb</td><td colspan="2">WBC</td><td colspan="2">Malaria Smear</td></tr>
<tr><td colspan="2"></td><td colspan="2"></td><td colspan="2"></td></tr>
</table>

| Other tests ordered and results: | |
|---|---|
| **12. Diagnoses This Admission:** | |
| 1. | 6. |
| 2. | 7. |
| 3. | 8. |
| 4. | 9. |
| 5. | 10. |

13. Drugs prescribed this admission:

| Date day/month/year | Drugs | Dose | Frequency | Duration |
|---|---|---|---|---|
| | | | | |
| | | | | |
| | | | | |
| | | | | |
| | | | | |
| | | | | |
| | | | | |
| | | | | |
| | | | | |
| | | | | |
| | | | | |

14. Daily Clinical Notes

| Date day/month/year (use as man | Observations | Temperature | *Write all medications given on the date given. You may require multiple lines for a given date. Some medications may have been prescribed in 13 which were not given due to stockouts and should not be included here)* |
|---|---|---|---|
| | | | |
| | | | |
| | | | |
| | | | |
| | | | |
| | | | |

| | | | |
|---|---|---|---|
| | | | |
| | | | |
| | | | |
| | | | |
| | | | |
| | | | |
| | | | |

please go to the continuation form if no room left on this document

15. Transfused this admission? ☐ No ☐ Yes
Date: ____/_____/________
day/month/year

16. Outcome this admission? ☐Discharged ☐Died ☐Unknown
Date of Discharge/Death: ____/_____/________
day/month/year

16. Counseled on HIV ☐ No ☐ Yes

17. Tested for HIV☐ No ☐ Yes

18. HIV results: ☐positive ☐negative ☐IND
Date: ____/_____/________
day/month/year

**Appendix 3: Linkage Form**
**Hospital-based Malaria Surveillance Linkage Form**

**Names of child____________________________________ Date ___/__/200_____**
**Father's name___________________________ Mother's name _______________________**

**LINKAGE TO PREVIOUS PARTICIPATION IN RP STUDIES**

1. Have you or this child ever lived in a household that any Rakai Project/RAIN staff ever:

| | Yes | No | DR |
|---|---|---|---|
| Conduct census/enumeration in a household you were residing in | 1 | 2 | 9 |
| Conducted census/enumeration in a household you were visiting or temporally residing. | 1 | 2 | 9 |
| Conduct census/enumeration or any research activities | 1 | 2 | 9 |
| Administered a questionnaire to you which included questions on health | 1 | 2 | 9 |
| Drawn blood from you or requested you to provide a blood sample for testing diseases. | 1 | 2 | 9 |
| Requested you to provide a urine sample/Swabs | 1 | 2 | 9 |
| Provided drugs to you for swallowing under direct observed therapy (whether you agreed to take them or not) | 1 | 2 | 9 |
| Visited a household where you resided and conducted research activities on all members of the household including children (these research activities included questionnaire administration, blood draw, physical examination, measuring body weight) | 1 | 2 | 9 |
| Taken an instant photo identification of you | 1 | 2 | 9 |
| Left you with a signed consent form showing your willingness to participate in an RP study. | 1 | 2 | 9 |

**IF YES TO ANY OF ABOVE FILL DETAILS; IF NO TO ALL SKIP TABLE AND CIRCLE FINAL DISPOSITION**

| Likely Study/studies | Region | Community | House hold head | Relation to head | other members in House Hold | Other info (e.g number of times seen, bled) |
|---|---|---|---|---|---|---|
| | | | | | | |

What names did you use when participating in the above mentioned activities? ____________________________________
FINAL DISPOSITION: ( AFTER CONSULTATION) |__| DISPO
**1** Not located in census database: give household current id # _______/_______/______/_____
**4** Not located in the census database
Names of child found in database___________________________________________

## References


1. Snow RW, Guerra CA, Noor AM, Myint HY, Hay SI, 2005. The global distribution of clinical episodes of Plasmodium falciparum malaria. Nature 434: 214-217.
2. Genton B, 2008. Malaria vaccines: a toy for travelers or a tool for eradication? Expert Rev Vaccines 7: 597-611.
3. Greenwood BM, Fidock DA, Kyle DE, Kappe SH, Alonso PL, Collins FH, Duffy PE, 2008. Malaria: progress, perils, and prospects for eradication. J. Clin. Invest. 118: 1266-76.
4. MVAC, 2006. Malaria Vaccine Technology Road Map. Malaria Vaccine Advisory Committee: WHO Initative for Vaccine Research.
5. Greenwood B, 2005. Interpreting vaccine efficacy. Clin. Infect. Dis. 40: 1519-1520.
6. Beier JC, Killeen GF, Githure JI, 1999. Short report: Entomologic inoculation rates and Plasmodium falciparum malaria prevalence in Africa. Am. J. Trop. Med. Hyg. 61: 109-113.
7. Guerra CA, Gikandi PW, Tatem AJ, Noor AM, Smith DL, Hay SI, Snow RW, 2008. The limits and intensity of Plasmodium falciparum transmission: implications for malaria control and elimination worldwide. PLoS Med 5: e38.
8. Greenwood B, 2005. Interpreting vaccine efficacy. Clin. Infect. Dis. 40: 1519-1520.
9. Moorthy VS, Reed Z, Smith PG, 2009. Clinical trials to estimate the efficacy of preventive interventions against malaria in paediatric populations: a methodological review. Malar J 8: 23.
10. Struchiner CJ, Halloran ME, Brunet RC, Ribeiro JM, Massad E, 1994. Malaria vaccines: lessons from field trials. Cad. Saude Publica 10 Suppl 2: 310-26.
11. O'Meara WP, Hall BF, McKenzie FE, 2007. Malaria vaccine efficacy: the difficulty of detecting and diagnosing malaria. Malar J 6: 36.
12. O'Meara WP, Lang T, 2009. Malaria vaccine trial endpoints – bridging the gaps between trial design, public health and the next generation of vaccines. Parasite Immunol. in press.
13. Hopkins H, Bebell L, Kambale W, Dokomajilar C, Rosenthal PJ, et al. (2008) Rapid diagnostic tests for malaria at sites of varying transmission intensity in Uganda. J Infect Dis 197: 510-518.]
