# Supplementary material for: High Prevalence of Malaria Parasitemia and Anemia among Hospitalized Children in Rakai, Uganda
Source: PLoS One. 2013 Dec 17;8(12):e82455. doi: 10.1371/journal.pone.0082455 (PMC3866122; doi:10.1371/journal.pone.0082455)
Supplement: Study Approval S1 — (ZIP) [file Study approvals S1.pdf › Study approvals S1.pdf]

Tel: (256) 41- 321962 (Direct)
(256) 41- 320385/6 (General)
Fax: (256) 41- 320483
Email:directoruvri@ug.cdc.gov

THE REPUBLIC OF UGANDA

UGANDA VIRUS RESEARCH INSTITUTE (UVRI)
P.O.Box 49 ENTEBBE (U)

Our Ref : GC/127/11/07/07
Your Ref:

15th July 2011

Dr. Valerian Kigundu,

RE: UVRI SEC expedited review of the protocol amendment titled **"RAKAI HEALTH FACILITY-BASED MALARIA OBSERVATIONAL STUDY"**

Thank you for your response to the queries addressed to you by UVRI SEC after the expedited review of the above study dated 12th July 2011 to the UVRI Science and Ethics Committee.

This is to inform you that your response was expeditely reviewed and found to have met the requirements of the UVRI Science and Ethics Committee.

UVRI SEC approval has been given for you to continue with your study with the proposed amendment. Annual progress report and request for extension should be submitted to UVRI SEC prior to the expiry date, to allow timely review.

The approved amendments include;

- Replacement of Dr.Noah Kiwanuka by Dr. Valerian Kigundu,
- Change of the title on the consent forms to read the same as the protocol.
- Provision to write the name of child and parent /guardian on the consent forms.
- Dropped Sahil`s. method for Hb estimation to hemacue to each centre.
- Page footer, updated to version 1.1 dated 13th June, 2011.
- Minor modifications made to the data capture forms.

You can now continue with your study after registration of your amendment with the Uganda National Council for Science and Technology (UNCST).

Yours sincerely,

Ahamale (NAMALE)

Dr. Alice Namale
Vice Chair, UVRI SEC

C.C Secretary, UVRI SEC

Uganda National Council for Science and Technology

(Established by Act of Parliament of the Republic of Uganda)

UNCST

Our Ref: HS 890 **September 8, 2011**

Dr. Valerian L. Kiggundu
Site Principal Investigator
Rakai Health Sciences Program
Kalisizo Station
RAKAI

Dear Dr. Kiggundu,

RE: RAKAI HEALTH FACILITY BASED MALARIA OBSERVATIONAL STUDY

This is to inform you that on 29 July 2011, Uganda National Council for Science and Technology (UNCST) reviewed and approved the amendment made to the above study.

The approved changes include:
1. Replacement of Dr. Noah Kiwanuka with Dr. Valerian Kiggundu.
2. Change of title on consent forms to read same as on the protocol document.
3. Provision to write the name of the child and parent/guardian on the Consent Forms.
4. Drop of the Sahil's method for Hb estimation to hemacue to each centre.
5. Update of page footer to Version 1.1 dated 13 June 2011.
6. Minor changes to the data capture forms.

Yours sincerely,

Jane Nabbuto
for: Executive Secretary
UGANDA NATIONAL COUNCIL FOR SCIENCE AND TECHNOLOGY

LOCATION/CORRESPONDENCE

Plot 6 Kimera Road, Ntinda
P. O. Box 6884
KAMPALA, UGANDA

COMMUNICATION

TEL: (256) 414 705500
FAX: (256) 414-234579
EMAIL: info@uncst.go.ug
WEBSITE: http://www.uncst.go.ug

IUPUI/CLARIAN INSTITUTIONAL REVIEW BOARD (IRB) REVIEW

APPLICATION FOR RESEARCH NOT SUBJECT TO FDA OR COMMON RULE DEFINITIONS OF HUMAN SUBJECTS RESEARCH

NS 1006-08

Please type only in the gray boxes. To mark a box as checked, double-click the box, select "checked", and click "OK".

SECTION I: PERSONNEL INFORMATION

Principal Investigator: Yiannoutsos, Constantin, T, PhD **Department:** Medicine

(Last, First, Middle Initial------must have faculty/staff status or faculty sponsor must sign)

Building/Room No.: HITS 3000 Phone: 278-3045 E-Mail: cyiannou@iupui.edu

Contact Information:

Name: Sheila Ellinger Address: BF 236 Phone: 278-4607

Fax: 278-2946 E-Mail: sellinge@iupui.edu

If this is a Student Protocol, List Name of the Student: ______ Phone: ______

Protocol Title: RAKAI HEALTH FACILITY-BASED MALARIA SURVEILLANCE STUDY

Sponsor/Funding Agency: NIAID, NICHD, NCI PI on Grant: Constantin Yiannoutsos

Sponsor Protocol #/Grant #: U01 NO6991 1-01 Period: From: 4/1/06 to 3/31/11

Sponsor Type: ☒ Federal ☐ State ☐ Industry ☐ Not-for-Profit ☐ Unfunded; ☐ Internally Funded

Grant Title (if different from project title): East Africa Regional IeDEA Consortium

SECTION II: PROJECT TYPE

Refer to the **Checklist for Determining Whether an Activity Requires Review by the IUPUI/Clarian IRB** for additional information.

☐ **Research Involving Data on Decedent PHI.** Please indicate that the following criteria are satisfied:

- ☐ The use is solely for research on the identifiable health information of decedents.
- ☐ The PHI sought is necessary for the purposes of the research; and
- ☐ Upon request, the covered entity disclosing the data may require the investigator to provide documentation of the death of the individual(s) about whom information is being sought.

☐ **Limited Data Set.** The research uses or discloses PHI as a limited data set for research purposes.

This project type may only be selected if the following is true: Your data set excludes 16 specified identifiers that are listed in the regulations, including: name, street address, telephone and fax numbers, e-mail address, social security number, certificate/license number, vehicle identifiers and serial numbers, URLs and IP addresses, and full face photos and other comparable images. The limited data set could include the following identifiable information: admission, discharge, and service dates, date of death, age (including age 90 and older), and five digit zip code.

Indicate from where the data will be obtained:

☐ The data will be provided from a covered entity (e.g. division, department, or practice plan) separate from that of the investigator. **NOTE: A data use agreement must be established between the entity(ies) providing the data and the investigator.** See the **Confidentiality and Privacy SOP** for additional information.

☐ The data will be obtained from within the investigator's own covered entity (e.g. his/her own data or that of the department). **No data use agreement is required.**

☐ Other, please explain:

☒ **De-Identified Health Information.** The research involves the use or disclosure of de-identified health information.

This project type may only be selected if the following is true: The health information excludes all of the following: (1) Name; (2) All geographic subdivisions smaller than a state, including street address, city, county, precinct, zip codes if the geographic unit of combining all the same three initial digits contains more than 20,000 people; (3) All elements of dates (except year) for dates directly related to an individual, including birth date, admission date, discharge date, date of death; and all ages over 89 and

all elements of dates (including year) indicative of such age, except that such ages and elements may be aggregated in a single category of age 90 or older; (4) Telephone numbers; (5) Fax numbers; (6) Electronic mail addresses; (7) Social security numbers; (8) Medical record numbers; (9) Health plan beneficiary numbers; (10) Account numbers; (11) Certificate/license numbers; (12) Vehicle identifiers and serial numbers, including license plate numbers; (13) Device identifiers and serial numbers; (14) Web universal resource locators (URLs); (15) Internet protocol (IP) address numbers; (16) Biometric identifiers, including finger and voice prints; (17) Full face photographic images and any comparable images; and (18) Any other unique identifying number, character, or code.

☐ **Coded Private Information or Biological Specimens.** The research involves only coded private information or specimens.

To qualify for this type of review, the private information or specimens cannot be linked to specific individuals by the investigator(s) either directly or indirectly through coding systems. To qualify, both of the following conditions must be met:

☐ The private information or specimens were **not** collected specifically for this proposed research project through an interaction or intervention with living individuals. **NOTE: If this condition is not met, then your research involves human subjects and requires a human subjects research submission.**

AND

☐ The investigator(s) cannot readily ascertain the identity of the individuals to whom the private information or specimens pertain because: (mark which option(s) applies)

☐ the key to decipher the code will be destroyed before the research begins
☐ the investigator(s) and the holder of the key will enter into an agreement prohibiting the release of the key to the investigator(s) under any circumstances, until the individuals are deceased
☐ Other. Please explain:______________________________

For additional information on research with coded private information or biological specimens, please refer to the OHRP Guidance on Research Involving Coded Private Information or Biological Specimens (October 16, 2008) at: http://www.hhs.gov/ohrp/humansubjects/guidance/cdebiol.pdf.

SECTION III: PROJECT DESCRIPTION

1. Provide a brief description, in lay terms, of the purpose of the proposed project and the procedures to be used.

We propose to conduct a hospital-based malaria surveillance study in Kalisizo and Bikira health center III in Rakai district, Uganda, to determine the proportion of patients with febrile illness that have malaria parasites on microscopy, and the proportions with severe malaria among children ≤5 years admitted to pediatric wards and malaria treatment outcomes among these children by HIV-1 serostatus.

The results of this study will improve the diagnosis and treatment of malaria cases in facilities and the management of non-malaria febrile illness in those who might have been diagnosed with malaria based on clinical symptoms in the absence of accurate testing. In addition, this study's results are expected to complement results from a community-based household surveillance study that will be conducted in communities of the Rakai Health Sciences Program cohort. Results from both studies will enhance the investigators' understanding of the epidemiology of pediatric and adult malaria infection in Rakai district in preparation for future malaria vaccine trials.

STUDY OBJECTIVES

1. To strengthen laboratory capacity and technical expertise at Kalisizo hospital and Bikira health center to provide routine parasitological confirmation of suspected malaria by microscopy and maintain a sensitivity and specificity of routine microscopy >90% as compared to reference microscopy.
2. To estimate the proportion of febrile illnesses with malaria parasites on a blood smear among all patients presenting to outpatient clinic at Kalisizo hospital
3. To estimate the prevalence, presentation, and treatment outcomes of severe malaria amongst HIV-infected and uninfected children aged <5 years admitted to Kalisizo and Bikira hospitals in Rakai district, Uganda.
4. To evaluate the feasibility of indentifying inpatient children with severe malaria that come from households within RCCS and linking them to RCCS database in order to assess the relationship between household and socio-demographic factors and the burden of malaria, and the progression to severe disease and death in HIV infected and uninfected children. (This objective will only be undertaken if additional funding is received from NIH for this objective)

2. Provide a list of all data points that will be collected below or attach a data collection sheet.

   1. De-identified data will be abstracted from OPD and lab record books from Kalisizo hospital to determine the number of patients who presented to OPD with febrile illness, number of patients with febrile illness who sent to the lab for malaria testing, and the number of febrile illness patients with positive malaria smear results.
   2. To estimate the prevalence, presentation, and treatment outcomes of malaria amongst HIV-infected and uninfected in-patient children, all children aged <5 years admitted to Kalisizo hospital and Bikira health center will be approached for enrollment into the study. A data collection sheet for inpatient use is included on page 14 of the protocol. All data will be collected by investigators in Uganda following local IREC approval. <u>All data will be totally de-identified per the definition of De-identified Health Information before sharing with Indiana University investigators</u>.
   3. To assess the relationship between household and socio-demographic factors collected during RCCS census activities and morbidity/mortality associated with severe malaria, the study will identify patients that come from RCCS censused households and link them to RCCS census database so as to obtain their household level census data. <u>Data shared for research purposes will be de-identified.</u>

Statement of Principal Investigator. By submitting this form, the Principal Investigator acknowledges that he/she has personally reviewed this report and agrees with the above assessment.

For IRB Use Only:

☒ Accepted

☐ Denied
- ☐ Separate human subjects application must be submitted.
- ☐ Project doesn't meet ethical principles
- ☐ Other action required:____________________

Authorized Signature: Regina Weber ____________________ Date: JUN 24 2010

Uganda National Council for Science and Technology
(Established by Act of Parliament of the Republic of Uganda)
UNCST

Our Ref: HS 890 **28/03/2011**

Dr. Noah Kiwanuka
Rakai Health Sciences Program
Uganda Virus Research Institute
P.O Box 49
Entebbe

Dear Dr. Kiwanuka,

RE: RESEARCH PROJECT, "FACILITY BASED MALARIA OBSERVATIONAL STUDY"

This is to inform you that the Uganda National Council for Science and Technology (UNCST) approved the above research proposal on **December 20, 2010.** The approval will expire on **December 20, 2011.** If it is necessary to continue with the research beyond the expiry date, a request for continuation should be made in writing to the Executive Secretary, UNCST.

Any problems of a serious nature related to the execution of your research project should be brought to the attention of the UNCST, and any changes to the research protocol should not be implemented without UNCST's approval except when necessary to eliminate apparent immediate hazards to the research participant(s).

This letter also serves as proof of UNCST approval and as a reminder for you to submit to UNCST timely progress reports and a final report on completion of the research project.

Yours sincerely,

Winfred Badanga
for: Executive Secretary
UGANDA NATIONAL COUNCIL FOR SCIENCE AND TECHNOLOGY

LOCATION/CORRESPONDENCE

Plot 6 Kimera Road, Ntinda
P. O. Box 6884

COMMUNICATION

TEL: (256) 414 705500
FAX: (256) 414-234579

Tel: (256) 41- 321962 (Direct)
(256) 41- 320385/6 (General)

Fax: (256) 41- 320483

Email:directoruvri@uvri.go.ug

THE REPUBLIC OF UGANDA

UGANDA VIRUS RESEARCH INSTITUTE (UVRI)
P.O.Box 49 ENTEBBE (U)

Our Ref: GC/127/11/10/17
Your Ref:

29th November 2010

Drs. Noah Kiwanuka, Yiannoutsos, et-al,

RE: UVRI SEC review of protocol titled **"Rakai –Health Facility – Based Malaria Surveillance study"**

Thank you for your response to the queries addressed to you during the SEC meeting of 21st October 2010.

This is to inform you that your responses dated 2nd November 2010 were reviewed during the SEC meeting of 25th November 2010 and met the requirements of the UVRI Science and Ethics Committee.

UVRI SEC approval has been given for you to conduct your research for one year up to 28th November 2011. Annual progress report and request for extension should be submitted to UVRI SEC two months prior to the expiry date, to allow timely review.

You can now commence with your study after registration with the Uganda National Council for Science and Technology (UNCST).

Wish you the best of luck.

Yours sincerely,

Dr. Alice Namale
Vice Chair, UVRI SEC

C.C Secretary, UVRI SEC
